# HIV self-testing for men who have sex with men in Sweden. A cross-sectional study concerning interest to use HIV self-tests

**DOI:** 10.1080/16549716.2021.2021631

**Published:** 2022-03-15

**Authors:** Elin Kinnman, Tobias Herder, Per Björkman, Fredrik Månsson, Anette Agardh

**Affiliations:** a Division of Social Medicine and Global Health, Department of Clinical Sciences, Lund University, Malmö, Sweden; b Clinical Infection Medicine, Department of Translational Medicine, Lund University, Malmö, Sweden

**Keywords:** HIVST, MSM, key populations, HIV prevention, HIV policy

## Abstract

**Background:**

HIV self-testing (HIVST) has been found to have high acceptability among men who have sex with men (MSM) internationally and might contribute to increase testing frequencies, but many countries, including Sweden, lack policies for using HIVST.

**Objective:**

To examine interest to use and willingness to pay for HIVST, and associated factors, among MSM attending HIV testing venues in Sweden.

**Method:**

This cross-sectional study analyzed data from a self-administered survey, consisting of 33 questions, collected at six HIV testing venues in Sweden in 2018. The sample consisted of sexually active men who have sex with men, aged ≥ 18 years, and not diagnosed with HIV. Data were analyzed descriptively and by univariable and multivariable logistic regression.

**Result:**

Among 663 participants (median age 33 years), 436 respondents (65.8%) expressed interest to use HIVST. Among those interested, less than half, 205 (47.0%), were willing to pay for HIVST. Being interested in HIVST was found to be negatively associated with being in the 55 years or older age group (AOR 0.31, CI 0.14–0.71), and having had syphilis, rectal chlamydia, or rectal gonorrhea in the preceding 12 months (AOR 0.56, CI 0.32–0.99). In the sample of MSM interested in HIVST, willingness to pay was positively associated with being in the age groups 35–44 years (AOR 2.94, CI 1.40–6.21), 45–54 years (AOR 2.82, CI 1.16–6.90), and 55 years or above (AOR 3.90, CI 1.19–12.81), and negatively associated with being single (AOR 0.56, CI 0.36–0.88).

**Conclusion:**

This study found high interest for HIVST in a sample of MSM in Sweden. However, HIVST offered at a cost is likely to negatively affect uptake among MSM broadly, compared with free availability.

## Background

The proportion of undiagnosed HIV infections in Sweden has been estimated to approximately 10%, and in 2016 Sweden was claimed to be the first country to reach the UNAIDS fast-track 90–90-90 targets [1]. The targets stated that by 2020, 90% of people living with HIV should know their status, among those who know their status 90% should be on treatment, and among those on treatment, 90% should achieve viral suppression. On the other hand, a more recent modeling study shows the uncertainties in estimations of proportions of undiagnosed HIV in Sweden, indicating that the true proportion is likely to be higher [2]. Further, late diagnosis of HIV is a global concern across high and low-income countries, and a study from Sweden found that 58% of people diagnosed with HIV were late presenters [3]. Increased testing could lead to earlier detection and treatment initiation, reducing HIV-related morbidity and mortality, in addition to curbing further transmission [4,5].

HIV self-testing (HIVST) allows users to test self-collected blood or saliva and, unassisted by health care professionals, interpret the results [6]. HIVST has been shown to be acceptable among various groups of people in diverse settings and is available in several countries [7–12]. The testing method has proven advantageous for key populations as it increases access, uptake, and frequency of HIV testing [11–14]. Among men who have sex with men (MSM) in Sweden, inconvenient clinic hours, concerns regarding confidentiality and long distances to testing venues have been highlighted as major barriers to testing [15], and HIVST could potentially overcome these.

The formal legal context differs between countries, with some countries fully allowing HIVST, others only partially authorizing HIVST for either sale, use, or distribution, and some (e.g. Singapore) making it explicitly illegal [16,17]. Further, other countries such as Sweden have no formal policies regarding HIVST [16]. Self-tests are nevertheless available also in countries without established formal policies and guidelines, for example, through online pharmacies. In Sweden, it is today possible to buy HIV self-test from online shops at a cost of approximately 25 USD. Related to this, WHO emphasize that this informal and unregulated access to HIV self-tests may give room for products of undetermined quality, performance, and safety [12], thus risking unreliable tests being available. Even if approved and recommended rapid diagnostic tests for HIVST have high sensitivity and specificity [12] one concern in low prevalence settings or populations would be the proportion of false-positive results [18]. Further, regulatory frameworks that can provide protection, monitoring, and facilitate reporting are central to safeguarding from misuse and abuse, such as potential unintended social harm in the form of coercive testing and discrimination [6,12,19]. Between 2015 and 2019, the number of countries with formal policies for HIVST has increased by nearly thirteen-fold, from 6 countries to 77. Further, between 2017 and 2019, the number of countries implementing HIVST programs has nearly tripled, from 14 to 38 [20]. In countries with broader availability of HIVST, such as Belarus, which was one of the first European countries to introduce HIVST for sales at pharmacies in 2017 [21], South Africa, and Kenya [22], tests are sold at pharmacies. This leads to availability for broader populations, but access and uptake depend on ability and willingness to pay. There have also been numerous examples where HIVST has been distributed for free in programs and trials specifically targeting key populations with increased risk for HIV infections, such as in Kenya among MSM [23] and among truck drivers and female sex workers [24]. WHO recommends HIVST to be offered as a supplement to existing HIV testing services [12], and the use of HIVST is included as part of the establishment of differentiated HIV testing strategies in the UN political declaration on HIV and AIDS [25].

As MSM account for a considerable proportion of HIV cases in Sweden [26], this group is essential to target when developing prevention strategies, including HIVST. In a statement from 2016, the Public Health Agency of Sweden advised against the use of HIVST, based on the argument that testing opportunities in the country are adequate and that HIVST leads to missed opportunities for counseling and testing for other STIs [27]. While previous studies and reports have investigated the prevention needs and preferences among Swedish MSM [15,28–33], no previous studies concerning self-test in the Swedish setting have been identified by the authors. A greater understanding of prevention preferences enables decision makers to make informed policy decisions. Based on this, the current study set out to examine the interest to use HIVST, as well as the willingness to pay for HIVST, and associated factors in a sample of MSM attending HIV testing venues in Sweden.

## Method

### Study design

This is a cross-sectional study based on data from a self-administered survey distributed to men attending one of six HIV/STI testing sites in Sweden’s three most populated cities: Stockholm, Gothenburg, and Malmö, between August and November 2018. For each city, one clinic within the public health-care system and one community-based rapid HIV testing venue were selected, resulting in a total of three health care and three community-based venues. The questionnaire, consisting of 33 questions, was piloted before data collection, and was available in Swedish and English. The data collection has been further described previously [30].

The inclusion criteria were as follows: identifying as a man, age ≥18 years, not diagnosed with HIV infection, and reporting anal or oral sex with another man during the preceding 12 months. Persons with missing data for the main dependent variable (HIVST) were excluded. Ethical approval for the study was obtained from the Regional Ethical Review Board in Lund, Sweden (dnr. 2017/687).

### Measures

All measures are based on self-reported survey data. An overview of the survey items and coding of included variables is available in a Supplementary table (online).

#### Dependent variables

The dependent variables, *Interest to use HIVST* and *Willingness to pay for HIVST*, were constructed from the question ‘Do you believe you will use a self-test for HIV in the future?’ with the three response options being ‘No’, ‘Yes, but only if it is free’, and ‘Yes, and I would be willing to pay for it’. For this study, the main dependent variable, Interest to use HIVST was defined as having answered either ‘Yes, but only if it is free’ or ‘Yes, and I would be willing to pay for it’. Willingness to pay for HIVST, which was used for further analysis, was defined as having answered: ‘Yes, and I would be willing to pay for it’.

#### Sociodemographic

*Age group* was categorized as 18–24 (reference category), 25–34, 35–44, 45–54, and 55 years or above. Education level was assessed by asking respondents about their highest education level and dichotomized as *University education*. Migrant background was defined as being *Born outside of Sweden*, and for those born in other countries, *New resident in Sweden* was defined as having lived in Sweden 5 years or less. *Non-heterosexual sexual orientation* was assessed by combining self-defined sexual orientations homosexual/gay, and bisexual/pansexual. Respondents were classified as *Being single* if respondents defined their relationship status as such.

#### Behavioral and HIV risk characteristics

Number of male sexual partners in the preceding 12 months was dichotomized based on the median value of the sample into *Seven partners or more* and categorized as yes or no. *Receptive condomless anal intercourse* with another man during the preceding 12 months was categorized as yes or no, based on respondent’s report. Use of intoxicants during sex was selected by respondents from a pre-defined list consisting of alcohol, amphetamine, cannabis, gamma-hydroxybutyrate (GHB) or gamma-butyrolactone (GBL), heroin, ketamine, cocaine, mephedrone, methylenedioxy-methamphetamine (MDMA) or ecstasy, methamphetamine/ice, poppers (alkyl nitrate inhalants), or other substance. Two variables regarding intoxicants were recoded for analysis; *Hard drug use during sex*, where alcohol, poppers, cannabis, and ‘other’ were excluded, and *Poppers use during sex*, motivated by the debated association between poppers use and behaviors associated with HIV and STI [34]. *Sex abroad* was assessed by asking the respondents if they had had sex outside Sweden during the previous 12 months, motivated by the high proportion of new HIV diagnoses in Sweden being contracted abroad [26]. STI is a well-recognized risk factor for HIV, which increases the biological likelihood of contracting HIV [35,36]. A combined variable for self-reported rectal gonorrhea, rectal chlamydia, and/or syphilis infection during the previous 12 months was constructed as *Combined risk STI*. The inclusion of this variable was based on the diagnoses highlighted as indicators of risk for seroconversion in the national PrEP recommendations [37]. Self-assessed HIV risk was included as *Moderate to high self-assessed HIV risk*, where responses of no or low risk were coded as no and moderate or high as yes.

#### Testing habits

Frequency of testing for HIV was assessed through two variables; *First-time tester*, indicating a response of never having been tested for HIV before, and *High frequency tester*, indicating the response of being tested twice a year or more. Further, the variables *Tested at NGO for HIV* in the last 12 months and having *Used a self-sampling kit for chlamydia and/or gonorrhea* in the last 12 months, *Ever having used a HIVST* were also examined.

### Statistical analysis

Descriptive statistics provided an overview of all included study participants. Pearson’s chi-square test was used to examine the distribution of covariates and test for differences in proportions between the strata in the descriptive analyses, with significance level set at a p- value of <0.05. An analysis of the descriptive results along with a literature review guided the selection of covariates for univariable and multivariable logistic regressions. An initial analysis of the main dependent variable was conducted, after which the sub-sample consisting of respondents interested in HIVST was further analyzed regarding willingness to pay for HIVST. Results are presented as crude odds ratios (OR) and adjusted odds ratios (AOR) with a 95% Confidence Interval (CI). Multicollinearity was considered by conducting variance inflation factor diagnosis, and goodness of fit for the final multivariable models were tested by Hosmer–Lemeshow test. Statistical analysis was performed using used Stata/SE 16.1 (StataCorp. 2019. College Station, TX: StataCorp LLC).

## Results

A total of 1672 men were invited to participate in the study, and 1351 surveys were returned. For the current study, data from 663 participants with a median age of 32 years (IQR 27–41) who met the inclusion criteria were analyzed. The majority of the included respondents, 514 (78.1%) self-identified as gay/homosexual, 123 (18.7%) as bisexual/pansexual, and 21 (3.2%) as heterosexual/straight. Seven individuals (1.1%) reported being assigned female sex at birth. Among the respondents, 403 (61.3%) reported having a university education, and 398 (60.3%) reported being single. While 240 (36.6%) reported being born outside of Sweden, 125 (19.3%) reported having lived in the country less than 5 years.

Frequencies of sociodemographic characteristics, risk characteristics for HIV, and testing habits are presented in Table 1, stratified by the main dependent variable, as well as willingness to pay for HIVST in the sub-sample of MSM interested in HIVST. The median number of male sexual partners in the preceding 12 months was 7 partners, and for further analysis this variable was dichotomized with a cut-off at 7 partners or more. Only 18 respondents (2.7%) had ever used HIVST, and due to the low frequency, the variable was excluded from further analysis. Among the respondents, 436 (65.8%) reported interest to use HIVST, and among those interested, 205 (47.0%) expressed willingness to pay for HIVST. Having had rectal chlamydia, rectal gonorrhea, or syphilis during the preceding 12 months was reported by 81 respondents (12.3%). Further, 44 respondents (6.7%) reported not having been tested for HIV previously, and 33 (5.0%) reported having used a self-sampling kit for chlamydia and/or gonorrhea.

**Table 1.** Frequencies of sociodemographics, HIV risk indicators and testing habits among study participants, stratified by interest to use HIVST and willingness to pay for HIVST. Total n=663

		**Total (N=663)**	**Interest to use HIVST (N=663)**			**Willingness to pay for HIVST (N = 436)**		
		n (%)	Not interested to use HIVST n (%) (n=227)	Interested to use HIVST n (%) (n=436)	χ^2^	Not willing to pay for HIVST n (%) (n=231)	Willing to pay for HIVST n (%) (n=205)	χ^2^
***Sociodemographics***								
**Age group**					**0.002**			**0.001**
	18-24	89 (13.4)	26 (11.5)	63 (14.5)		45 (19.5)	18 (8.8)	
	25-34	293 (44.2)	95 (41.9)	198 (45.4)		111 (48.1)	87 (42.4)	
	35-44	150 (22.6)	43 (18.9)	107 (24.5)		49 (21.2)	58 (28.3)	
	45-54	79 (11.9)	34 (15.0)	45 (10.3)		18 (7.8)	27 (13.2)	
	≥ 55	52 (7.84)	29 (12.8)	23 (5.3)		8 (3.5)	15 (7.3)	
	(missing)	0				0		
**University education**					0.737			0.096
	No	254 (38.7)	85 (37.8)	169 (39.1)		98 (42.8)	71 (35.0)	
	Yes	403 (61.3)	140 (62.2)	263 (60.9)		131 (57.2)	132 (65.0)	
	(missing)	6				4		
**Born outside Sweden**					0.067			0.754
	No	415 (63.4)	152 (68.2)	263 (60.9)		141 (61.6)	122 (60.1)	
	Yes	240 (36.7)	71 (31.8)	169 (39.1)		88 (38.4)	81 (39.9)	
	(missing)	8				4		
**New residents in Sweden,**					**0.042**			0.205
**lived in Sweden up to 5 years**								
	No	522 (80.7)	188 (85.1)	334 (78.4)		181 (80.8)	153 (75.7)	
	Yes	125 (19.3)	33 (14.9)	92 (21.6)		43 (19.2)	49 (24.3)	
	(missing)	16				10		
**Non-heterosexual sexual orientation**					0.690			0.628
	No	21 (3.2)	8 (3.6)	13 (3.0)		6 (2.6)	7 (3.4)	
	Yes	637 (96.8)	216 (96.4)	421 (97.0)		223 (97.4)	198 (96.6)	
	(missing)	5				2		
**Being single**					0.435			**<0.001**
	No	262 (39.7)	94 (41.8)	168 (38.6)		69 (30.0)	99 (48.3)	
	Yes	398 (60.3)	131 (58.2)	267 (61.4)		161 (70.0)	106 (51.7)	
	(missing)	3				1		
***Risk characteristics for HIV***								
**Seven partners or more, 12 months**					0.574			0.103
	No	315 (49.4)	111 (50.9)	204 (48.6)		99 (44.8)	105 (52.8)	
	Yes	323 (50.6)	107 (49.1)	216 (51.4)		122 (55.2)	94 (47.2)	
	(Missing)	25				16		
**Receptive condomless anal intercourse, 12 months**					0.836			0.128
	No	303 (45.7)	105 (46.3)	198 (45.4)		97 (42)	101 (49.3)	
	Yes	360 (54.3)	122 (53.7)	238 (54.6)		134 (58)	104 (50.7)	
	(Missing)	0				0		
**Hard drug use during sex, 12 months ^a^**					0.454			0.078
	No	563 (85.7)	196 (87.1)	367 (85)		188 (82.1)	179 (88.2)	
	Yes	94 (14.3)	29 (12.9)	65 (15)		41 (17.9)	24 (11.8)	
	(Missing)	6				4		
**Poppers use during sex, 12 months**					0.221			0.886
	No	426 (64.8)	153 (68.0)	273 (63.2)		144 (62.9)	129 (63.5)	
	Yes	231 (35.2)	72 (32.0)	159 (36.8)		85 (37.1)	74 (36.5)	
	(Missing)	6				4		
**Combined risk STI, 12 months ^b^**					0.307			**0.001**
	No	575 (87.7)	194 (85.8)	381 (88.6)		193 (83.9)	188 (94.0)	
	Yes	81 (12.3)	32 (14.2)	49 (11.4)		37 (16.1)	12 (6.0)	
	(Missing)	7				6		
**Sex abroad, 12 months**					**0.020**			0.635
	No	260 (39.3)	103 (45.4)	157 (36.1)		81 (35.1)	76 (37.3)	
	Yes	402 (60.7)	124 (54.6)	278 (63.9)		150 (64.9)	128 (62.7)	
	(Missing)	1				1		
**Moderate to high self-assessed HIV risk**					0.929			0.726
	No	558 (85.1)	191 (84.9)	367 (85.2)		192 (84.6)	175 (85.8)	
	Yes	98 (14.9)	34 (15.1)	64 (14.8)		35 (15.4)	29 (14.2)	
	(Missing)	7				5		
***Testing habits***								
**First-time tester**					0.186			0.361
	No	615 (93.3)	214 (95.1)	401 (92.4)		210 (91.3)	191 (93.6)	
	Yes	44 (6.7)	11 (4.9)	33 (7.6)		20 (8.7)	13 (6.4)	
	(Missing)	4				2		
**High frequency tester, twice a year or more**					0.826			**0.018**
	No	288 (43.7)	97 (43.1)	191 (44)		89 (38.7)	102 (50.0)	
	Yes	371 (56.3)	128 (56.9)	243 (56)		141 (61.3)	102 (50.0)	
	(Missing)	4				2		
**Used a self-sampling kit for chlamydia and/or gonorrhea, 12 months**					**0.046**			0.500
	No	630 (95.0)	221 (97.4)	409 (93.8)		215 (93.1)	194 (94.6)	
	Yes	33 (5.0)	6 (2.6)	27 (6.2)		16 (6.9)	11 (5.4)	
	(Missing)	0				0		
**Tested at NGO, 12 months**					0.335			0.099
	No	392 (59.1)	140 (61.7)	252 (57.8)		142 (61.5)	110 (53.7)	
	Yes	271 (40.9)	87 (38.3)	184 (42.2)		89 (38.5)	95 (46.3)	
	(Missing)	0				0		

P-value was produced by Chi-square test (χ2). Bold font indicates statistical significance (p-value <0.05) a. Amphetamine, GHB/GBL, heroin, ketamine, cocaine, mephedrone, MDMA/ecstasy, methamphetamine/ice (during sex, 12 months).b. Rectal chlamydia, rectal gonorrhea or syphilis (12 months).

### Variables associated with interest in using HIVST

The results of the univariable logistic regression analysis showed statistically significant unadjusted associations between interest to use HIVST as illustrated in Table 2. A negative association was found between being interested in using HIVST and being in the age group 55 years or above (OR 0.32, CI 0.16–0.66), as compared to being in the 18–24 age group. Further, the odds of being interested in HIVST were higher if having lived in Sweden for ≤5 years (OR 1.57, CI 1.01–2.43). Additionally, those reporting having had sex abroad had higher odds of being interested in HIVST (OR 1.47, CI 1.06–2.04).

**Table 2.** Univariable and Multivariable logistic regression for interest to use HIVST among the study population. (N=663)

		**Univariable analysis**		Multivariable analysis*	
** **		**Crude OR (CI 95%)**		**Adjusted OR (CI 95%)**	
***Sociodemographics***					
**Age group**					
	18-24	ref		ref	
	25-34	0.86	(0.51-1.44)	0.76	(0.43-1.34)
	35-44	1.02	(0.58-1.83)	1.00	(0.53-1.89)
	45-54	0.55	(0.29-1.03)	0.57	(0.28-1.15)
	≥ 55	**0.32**	**(0.16-0.66)**	**0.31**	**(0.14-0.71)**
**University education**					
	No	ref		ref	
	Yes	0.94	(0.68-1.32)	0.90	(0.61-1.33)
**Born outside Sweden**					
	No	ref			
	Yes	1.38	(0.98-1.94)		
**New residents in Sweden, lived in Sweden up to 5 years**					
	No	ref	** **	ref	
	Yes	**1.57**	**(1.01-2.43)**	1.43	(0.86-2.37)
**Non-heterosexual sexual orientation**					
	No	ref		ref	
	Yes	1.20	(0.49-2.34)	0.91	(0.33-2.52)
**Being single**					
	No	ref		ref	
	Yes	1.14	(0.82-1.58)	1.03	(0.71-1.49)
***Risk characteristics for HIV***					
**Seven partners or more, 12 months**					
	No	ref		ref	
	Yes	1.10	(0.79-1.52)	0.98	(0.66-1.45)
**Receptive condomless anal intercourse, 12 months**					
	No	ref		ref	
	Yes	1.03	(0.75-1.43)	1.26	(0.86-1.85)
**Hard drug use during sex, 12 months ^a^**					
	No	ref		ref	
	Yes	1.20	(0.75-1.92)	1.12	(0.65-1.91)
**Poppers use during sex, 12 months**					
	No	ref			
	Yes	1.23	(0.88-1.74)		
**Combined risk STI, 12 months ^b^**					
	No	ref		ref	** **
	Yes	0.78	(0.48-1.26)	**0.56**	**(0.32-0.99)**
**Sex abroad, 12 months**					
	No	ref	** **	ref	
	Yes	**1.47**	**(1.06-2.04)**	1.37	(0.94-2.01)
**Moderate to high self-assessed HIV risk**		** **	** **	** **	** **
	No	ref		ref	
	Yes	0.98	(0.62-1.54)	1.01	(0.61-1.68)
***Testing habits***					
**First-time tester**					
	No	ref			
	Yes	1.60	(0.79-3.23)		
**High frequency tester, twice a year or more**					
	No	ref		ref	
	Yes	0.96	(0.70-1.33)	0.87	(0.59-1.28)
**Used a self-sampling kit for chlamydia and/or gonorrhea, 12 months**					
	No	ref			
	Yes	2.43	(0.99-5.98)		
**Tested at NGO, 12 months**					
	No	ref			
	Yes	1.17	(0.84-1.63)		

* For the multivariable analysis, all included variables in the analysis were mutually adjusted for each other and for venue. Bold font indicates statistical significance, CI 95%. a. Amphetamine, GHB/GBL, heroin, ketamine, cocaine, mephedrone, MDMA/ecstasy, methamphetamine/ice (during sex, 12 months). b. Rectal chlamydia, rectal gonorrhea or syphilis (12 months). P-value for goodness-of-fit of multivariable model (Hosmer-Lemeshow): 0.421

The multivariable logistic regression analysis for interest to use HIVST confirmed an independent negative association between being interested in using HIVST and being in the age group 55 years or above (AOR 0.31, CI 0.14–0.71) compared to being in the 18–24 age group. However, the analysis did not confirm the association between being a new resident in Sweden or having had sex abroad and interest in using HIVST. In addition, the analysis found a negative association between reporting a combined risk STI in the last 12 months and interest in using HIVST (AOR 0.56 CI 0.32–0.99).

In the further analysis of the sample of MSM who were interested in HIVST (N = 436), the univariable regression showed associations between willingness to pay and age, being single, combined risk STI, and being a high-frequency tester, as illustrated in Table 3. After adjustment in the multivariable model the results showed that being in the age groups 35–44 years (AOR 2.94, CI 1.40–6.21), 45–54 years (AOR 2.82, CI 1.16–6.90), and 55 years or above (AOR 3.90, CI 1.19–12.81) was positively associated with being willing to pay for HIVST in comparison to being 18–24 years old. Being single was found to be negatively associated with willingness to pay (AOR 0.56, CI 0.36–0.88).

**Table 3.** Univariable and Multivariable logistic regression for willingness to pay for HIVST among those interested in using HIVST. (N = 436)

		Univariable analysis		Multivariable analysis*	
		Crude OR (CI 95%)		Adjusted OR (CI 95%)	
***Sociodemographics***					
**Age group**					
	18–24	ref		ref	
	25–34	**1.96**	**(1.05–3.62)**	1.73	(0.89–3.36)
	35–44	**2.96**	**(1.52–5.75)**	**2.94**	**(1.40–6.21)**
	45–54	**3.75**	**(1.67–8.42)**	**2.82**	**(1.16–6.90)**
	≥ 55	**4.69**	**(1.69–12.97)**	**3.90**	**(1.19–12.81)**
**University education**					
	No	ref		ref	
	Yes	1.39	(0.94–2.05)	1.06	(0.65–1.71)
**Born outside Sweden**					
	No	ref			
	Yes	1.06	(0.72–1.56)		
**New residents in Sweden, lived in Sweden up to 5 years**					
	No	ref		ref	
	Yes	1.35	(0.85–2.14)	1.31	(0.73–2.34)
**Non-heterosexual sexual orientation**					
	No	ref		ref	
	Yes	0.76	(0.25–2.30)	0.91	(0.26–3.19)
**Being single**					
	No	ref		ref	
	Yes	**0.45**	**(0.31–0.68)**	**0.56**	**(0.36–0.88)**
***Risk characteristics for HIV***					
**Seven partners or more, 12 months**					
	No	ref		ref	
	Yes	0.73	(0.49–1.07)	0.90	(0.56–1.45)
**Receptive condomless anal intercourse, 12 months**					
	No	ref		ref	
	Yes	0.75	(0.51–1.09)	0.83	(0.52–1.34)
**Hard drug use during sex, 12 months ^a^**					
	No	ref		ref	
	Yes	0.61	(0.36–1.06)	0.90	(0.48–1.70)
**Poppers use during sex, 12 months**					
	No	ref			
	Yes	0.97	(0.66–1.44)		
**Combined risk STI, 12 months ^b^**					
	No	ref		ref	
	Yes	**0.33**	**(0.17–0.66)**	0.45	(0.20–1.02)
**Sex abroad, 12 months**					
	No	ref		ref	
	Yes	0.91	(0.61–1.35)	0.91	(0.56–1.47)
**Moderate to high self-assessed HIV risk**					
	No	ref		ref	
	Yes	0.91	(0.53–1.55)	0.98	(0.52–1.83)
***Testing habits***					
**First-time tester**					
	No	ref			
	Yes	0.71	(0.35–1.48)		
**High frequency tester, twice a year or more**					
	No	ref		ref	
	Yes	**0.63**	**(0.43–0.92)**	0.71	(0.45–1.13)
**Used a self-sampling kit for chlamydia and/or gonorrhea, 12 months**					
	No	ref			
	Yes	0.76	(0.35–1.68)		
**Tested at NGO, 12 months**					
	No	ref			
	Yes	1.37	(0.94–2.02)		

* For the multivariable analysis, all included variables in the analysis were mutually adjusted for each other and for venue. Bold font indicates statistical significance, CI 95%. a. Amphetamine, GHB/GBL, heroin, ketamine, cocaine, mephedrone, MDMA/ecstasy, methamphetamine/ice (during sex, 12 months). b. Rectal chlamydia, rectal gonorrhea or syphilis (12 months). P-value for goodness-of-fit of multivariable model (Hosmer-Lemeshow): 0.657

## Discussion

In this study, we found that a majority of the MSM visiting public and community-based HIV testing venues in three cities were interested in using HIVST. The overall pattern of few variables indicating significant associations with interest to use is a noteworthy finding on its own, demonstrating wide acceptability of the strategy, independent of sociodemographic or behavioral factors. However, as the willingness to pay for HIVST among the study population was considerably lower than the interest to use if provided for free, HIVST would likely be cost-prohibitive if introduced with a fee. Thus, it would risk leaving out nearly half of the individuals who would otherwise be interested in utilizing HIVST.

The high interest among MSM shown in our study is in line with findings among MSM in low and middle-income countries [38–41] as well as in other high-income countries [42–44]. This finding is also in line with results from a previous survey of the general population in Sweden, in which 62% of respondents expressed interest in HIVST [45]. In a systematic review of HIVST among key populations, including MSM, eight out of 14 studies reporting on acceptability found high acceptability of HIVST (≥67%), five studies found moderate acceptability (66–34%), and one study found acceptability to be low [7]. Further, Hoyos et al. found HIVST to be the preferred testing option for one-third of an online sample of MSM recruited in five European countries [46].

This study found a significant negative association between reporting having had a combined risk STI in the last 12 months and interest in HIVST. Since these STIs are known risk factors for HIV [35,36], this result is of particular interest as it indicates lower interest among MSM with increased risk of STI and HIV. It is also a relevant finding as one concern regarding HIVST has been the possibility of a decrease in STI testing, even though the evidence concerning this has been inconclusive [12–14]. Thus, it is important to further evaluate the potential impact of HIVST on STI testing in different contexts, along with other concerns such as HIVST not being used as intended to test oneself, but to screen potential sex partners, HIVST decreasing condom use, and HIVST increasing the risk of coerced HIV testing [7,14,47]. The potential concerns of medical, social, and psychological harm as well as ethical and legal issues underscore the need for policies, regulatory frameworks, and monitoring.

While the overall interest to use HIVST was high, among those interested, less than half were willing to pay for such tests. The potential negative effects on utilization of HIVST if introduced at cost have also been discussed in previous research [6,48,49]. The current study did not include levels of cost for HIVST for the respondents to consider, but a number of previous papers have examined this among MSM in different settings such as the US [50], Australia [51], and Spain [42]. It should be noted that HIV testing is provided free of cost in Sweden at any clinic, in accordance with the Swedish Communicable Diseases Act. This is not unique to the Swedish context, and HIV tests are commonly offered free of charge globally. In countries where HIV tests come with a fee, it is not uncommon with initiatives of free testing for key populations, such as MSM. This availability of free HIV testing might influence the willingness to pay for alternative HIV testing, such as HIVST. Nevertheless, the positive association between age and willingness to pay might indicate that socioeconomic position would affect uptake if HIVST was introduced with a cost. In the analyses, no clear association between risk characteristics and willingness to pay for HIVST could be established, which can be interpreted as cost being a possible barrier to HIVST across sub-groups of MSM, categorized according to HIV risk characteristics. Further studies on cost levels’ effect on HIV testing frequency are needed to determine whether cost level disincentivizes higher frequency of testing, as well as if different groups are affected differently by cost level.

HIVST has been highlighted as a strategy to reach people who for different reasons do not test for HIV, undertested individuals, and people who have never had an HIV test [12,52]. While the sampling strategy of this study made it impossible to include non-testers, variables assessing first-time and high-frequency testers could be examined. However, due to low frequency of first-time testers and no significance of association, this variable was not included in the multivariable analysis. With regard to high-frequency testers, no independent associations were found in multivariable analyses. This result is not entirely consistent with research from other settings. For example, a higher interest for HIVST was reported among sub-optimal and never-testers compared to optimal testers among gay and bisexual men in Australia [51]. Similarly, a French study found associations between being more interested if being a non-tester or having been tested more than 1 year ago [43]. While the studies highlighted above come from comparable countries, it is essential to note that knowledge and regulations on HIVST in the different settings vary, and access to free HIVST differs across settings and populations. While no independent association between having a migrant background and interest in HIVST could be established in this study, a recent study suggests that testing frequency in the group is comparatively higher but concludes that there is a need to further promote HIV testing among migrant MSM. When exploring preferences in HIV preventive services, rapid HIV testing and testing outside of the healthcare settings were two of the most requested services [33], findings that together with the results of this study suggest that HIVST might be a suitable strategy for this group.

It has been argued that the ultimate utility of HIVST will be realized when it is a part of a comprehensive approach (e.g. [51,53,54],). Although the Public Health Agency of Sweden has argued that testing opportunities are adequate [27], late diagnosis remains a public health concern [3]. Following the 90–90-90 targets that were set to be reached by 2020, lowering the proportion of people living with undiagnosed HIV is a continued priority for reaching the current UNAIDS fast-track 95–95-95 targets to be reached by 2030, a priority reiterated in the 2021 UN Political Declaration HIV and AIDS [25]. Innovative measures are necessary to reach the targets by 2030, and the declaration emphasizes the use of multiple testing technologies and approaches, including HIVST [25]. Further, by incorporating HIVST into the national strategy, follow-up strategies for both reactive and non-reactive results can be developed. For reactive results, referral and access to health-care facilities for confirmatory tests and linkages to care are essential. Ensuring successful referral poses a bigger challenge for HIVST than for venue-based testing, as the test is conducted without the supervision of trained personnel. Although reviews have found HIVST to have similar rates of linkage to care compared to venue-based testing [13,55], some researchers have questioned the generalizability of these results outside of the research/trial environments [56,57]. A clear policy inclusion of HIVST increases the possibilities of establishing a sound structure for linkages.

The majority of HIVST taken will yield a non-reactive result. By incorporating HIVST into the national HIV prevention strategy, opportunities to utilize these non-reactive tests as a gateway to biomedical and behavioral HIV/STI interventions increase. HIVST could, for example, be linked to Pre-Exposure Prophylaxis (PrEP) services by linking individuals with a non-reactive test but substantial risk of HIV acquisition to available services. Further, HIVST has been suggested to support PrEP rollout by streamlining it and reducing follow-up clinic visits by replacing some clinic-based testing with HIVST, contributing to a reduction of the burden on the health-care system [53,58]. Since individuals testing for HIV are likely to benefit from testing for other STIs, HIVST should also be used as a gateway to additional STI testing. How these interventions and linkages to services are best organized in relation to HIVST needs to be further studied, but one potential strategy is implementing HIVST by linking it to existing telemedicine or e-health services, where available, or specially developed digital applications and services. A recent review concluded that HIVST with digital support could improve efficiency of HIVST interventions and is suitable for at-risk populations [59]. In the literature, there are also specific examples of programs offering HIVST supervised via video chat [60,61], and in Brazil telemedicine was utilized for PrEP in combination with HIVST during Covid-19 [62].

Implementing HIVST might also contribute to ensuring access to testing when disruptions affect HIV testing services, which has been observed during the Covid-19 pandemic [62]. As the pandemic affected clinic hours and caused geographic isolation and reprioritization of staff tasks [63], it likely negatively affected access to and utilization of venue-based HIV testing services. The number of HIV tests conducted at public health centers in Japan significantly declined during Covid-19 [64], and in Melbourne, a 31% decrease in the number of tests was observed [65]. The same was observed in parts of the US, with a 40% decline in San Francisco and a 85% decline in Boston [66]. Similar trends could be seen among four of the six HIV testing venues participating in the data collection for this study, with substantial (27–45%) declines in the number of visits or conducted HIV tests 2020, when compared to 2019. Two of the venues did not have declines in HIV tests conducted. While the full effect of the pandemic on HIV prevention and services is yet to be determined, it has been argued that HIVST as a strategy can help to ease the strain on a burdened health-care system [67].

Since this is a cross-sectional study, it has inherent limitations regarding inferring causation [68]. The study’s essential strength was a carefully constructed questionnaire, structured to minimize potential misunderstandings and recall bias. The self-administered questionnaires and assurance of anonymity of responses are expected to limit social desirability bias in participants’ responses, as the survey included topics that can be considered to be sensitive [69]. A limitation regarding the questionnaire that needs to be considered is however the lack of information on acceptability of cost levels of HIVST. Not specifying cost in the question might lead more respondents to choose the ‘free’ option, than if the questionnaire had provided a number of specific cost levels among which they could choose from.

The limited representativeness of the study should be noted as the questionnaire was solely distributed at MSM testing venues in major urban cities. A population-based study would likely not have resulted in a sufficient sample size for the intended analysis, which specifically targeted MSM. The kind of facility-based sampling used here is one of the more commonly used strategies [70]. While an alternative approach, internet-based sampling, has been used in previous studies in Sweden, it has other limitations regarding capturing representative samples [15,71,72]. Facility-based sampling reaches an MSM population with high relevance for HIV prevention, and by including community-based venues first-time testers were reached to a greater extent [32]. Nevertheless, this sampling strategy fails to capture the experiences of non-testers, and in a previous study on MSM in Sweden this group comprised 23.6% of the sample [15]. Another group not fully captured are non-urban residents, and with limited testing opportunities in non-urban settings [31], one could hypothesize that interest for HIVST would be even greater in rural areas. As the sampling occurred at one STI clinic and one community-based testing venue in each city, some selection biases might have been introduced due to the varied opportunities for testing in the three cities. In order to limit the effects of this bias we controlled for venue in the multivariable logistic regression analyses. Young MSM are, for example likely to attend youth clinics, and other groups may have lower access, such as certain groups of migrants, and might thus be underrepresented in the sample. As high-frequency testers attend the venues more often, a certain overrepresentation is likely, which might lead to overestimations of risk behaviors. Thus, the results are to be interpreted with caution and may not be applicable to all MSM in Sweden. With these limitations in mind, the study nonetheless contributes with important new knowledge for the future HIV preventive work in Sweden and similar countries. Further studies are needed for greater understanding of HIVST as a possible strategy to reach other populations with increased risk for HIV in Sweden or undertested populations with high proportions of late presenters.

## Conclusion

This study showed that interest in HIVST varied with age among MSM who visit HIV testing venues but found limited support for variations in interest based on HIV risk characteristics. In the further analysis, we could not establish any independent associations between HIV risk characteristics and willingness to pay for HIVST. This finding suggests that if HIVST are introduced at a cost, it is likely that a fewer MSM, regardless of HIV risk characteristics, would utilize HIVST in comparison to if it was introduced without cost. However, considering the broad interest shown among MSM to use HIVST and the potential it has to overcome existing barriers to testing, HIVST could be a beneficial complement to existing HIV prevention programs for MSM in Sweden, as well as in other countries.

## Supplementary Material

Supplemental Material

## Data Availability

Due to the nature of this research, participants in this study did not agree for their data to be shared publicly, so supporting data is not available.

## Paper context

HIVST has been highlighted as a strategy to increase HIV testing uptake and frequency. However, there is limited information about HIVST in the Swedish setting. This study aimed to explore the interest to use and willingness to pay for HIVST among MSM in Sweden. Our results indicate that HIVST has the potential to be a positive addition to Sweden’s national HIV strategy and that there is a need for further research.
